# Evaluation of Modern Techniques for Species Identification of *Lutzia* Mosquitoes (Diptera: Culicidae) in Thailand: Geometric Morphometrics and DNA Barcoding

**DOI:** 10.3390/insects14010078

**Published:** 2023-01-12

**Authors:** Sedthapong Laojun, Tanasak Changbunjong, Tanawat Chaiphongpachara

**Affiliations:** 1 Department of Public Health and Health Promotion, College of Allied Health Sciences, Suan Sunandha Rajabhat University, Samut Songkhram 75000, Thailand; 2 Department of Pre-Clinic and Applied Animal Science, Faculty of Veterinary Science, Mahidol University, Nakhon Pathom 73170, Thailand; 3 The Monitoring and Surveillance Center for Zoonotic Diseases in Wildlife and Exotic Animals (MoZWE), Faculty of Veterinary Science, Mahidol University, Nakhon Pathom 73170, Thailand

**Keywords:** mosquito, *Lutzia*, geometric morphometrics, DNA barcoding, Thailand

## Abstract

**Simple Summary:**

There are four species of *Lutzia* mosquitoes in Thailand, including *Lt. chiangmaiensis*, *Lt. fuscana*, *Lt. halifaxii*, and *Lt. vorax*. However, it is difficult to identify damaged specimens when some of their morphological character is missing. Thus, we evaluated the efficacy of the geometric morphometric (GM) approach and DNA barcoding for the identification of four *Lutzia* species. Our results showed that DNA barcoding is poorly effective in identifying *Lt. fuscana* and *Lt. halifaxii* based on their low interspecific genetic differences. On the other hand, the GM approach based on wing shape analyses successfully identified the four *Lutzia* species in Thailand.

**Abstract:**

There are four species of *Lutzia* mosquitoes in Thailand, including *Lutzia chiangmaiensis*, *Lt. fuscana*, *Lt. halifaxii*, and *Lt. vorax.* The accurate species identification of adult *Lutzia* mosquitoes based on morphological features requires many body parts, including the abdominal terga and wing. However, species identification is difficult in the case of damaged specimens when some of their morphological character is missing due to transit or gathering in the field. Thus, we evaluated the efficacy of the landmark-based geometric morphometric (GM) approach for the discrimination of *Lutzia* species in Thailand. In addition, DNA barcoding was also used in parallel with the GM approach to identify the species. Larvae of *Lutzia* were collected, raised into adults, and identified based on their morphological characteristics. The validated reclassification test results clearly demonstrated that wing shape resulted in a high level of success in identification (correct identifications ranged from 92.50% to 100%); however, based on the DNA barcoding analyses, our results showed that it was poorly effective in identifying *Lt. fuscana* and *Lt. halifaxii* based on an overlap between the intraspecific and interspecific divergence. Moreover, our survey results provide updates on the distribution of *Lt. chiangmaiensis* and *Lt. vorax* in Thailand. This research will help medical entomologists more efficiently identify mosquitoes in the genus *Lutzia*, resulting in more effective mosquito control and surveillance.

## 1. Introduction

*Lutzia* are large mosquitoes that belong to the order Diptera, subfamily Culicidae, tribe Culicini. This genus consists of nine formally recognized species divided into three subgenera, including *Insulalutzia* (one species: *Lt. shinonagai*), *Lutzia* (two species: *Lt. allostigma* and *Lt. bigoti*), and *Metalutzia* (six species: *Lt. agranensis*, *Lt. chiangmaiensi*, *Lt. fuscana*, *Lt. halifaxii*, *Lt. tigripes*, and *Lt. vorax*) [1]. Recently, phylogenetic analyses based on DNA sequence data on the first and second internal transcribed spacer (ITS-1 and ITS-2) regions of nuclear ribosomal DNA (rDNA) and the mitochondrial cytochrome *c* oxidase I (mtDNA-*COI*) revealed that the *Lutzia* clade is classified as a species of the subgenera of *Culex* [2,3]. However, *Lutzia* is currently classified as a distinct genus from the genus *Culex* based on its fundamentally distinctive morphological and biological identity [1].

*Lutzia shinonagai*, the only species of the subgenus *Insulalutzia*, is exclusively found on Ogasawara Island, Japan, whereas *Lt. allostigma* and *Lt. bigoti*, two species of the subgenus *Lutzia*, are distributed in the Neotropical region. Some species of the subgenus *Metalutzia* have limited distribution: *Lt. agranensis* is reported only in India; *Lt. chiangmaiensis* is reported only in northern Thailand. Other species of this subgenus are widely distributed: *Lt. fuscana* and *Lt. halifaxii* are distributed in the Oriental, Australian, and eastern Palearctic regions; *Lt. tigripes* is distributed throughout the Afrotropical region; and *Lt. vorax* is distributed in the Oriental and Australian regions [1,4].

Mosquito species in the genus *Lutzia* have not been reported to carry pathogens of human diseases because female *Lutzia* mosquitoes feed mainly on the blood of mammals and birds and seldom attack humans [5]. However, *Lutzia* mosquitoes serve as natural biological controls on mosquito vectors, making them inevitably important for public health. *Lutzia* larvae are voracious predators that feed primarily on the larvae of other mosquito species. Surendran et al. [6] assessed the effectiveness of *Lutzia* larvae as a predator on mosquito vectors in Sri Lanka and found that they were most effective at hunting *Aedes aegypti* larvae. The immature stages of *Lutzia* are typically found in a wide variety of freshwater habitats, which are similar to those occupied by *Culex* species, and have been found in wheel ruts and barrels, which are similar to those occupied by *Aedes* species, and are most probably related to their desired prey [1].

There are four different species of *Lutzia* mosquitoes in Thailand, including *Lt. chiangmaiensis*, *Lt. fuscana*, *Lt. halifaxii*, and *Lt. vorax* [4,7]. Rattanarithikul et al. [5] reported that *Lt. fuscana* and *Lt. halifaxii* are distributed across Thailand, whereas *Lt. vorax* are only distributed in the northern, western, and central regions. Lately, Somboon and Harbach [7] reported that *Lt. chiangmaiensis* is distributed in northern Thailand, while its presence in other regions remains unconfirmed. According to the gold standard, the species identification of *Lutzia* mosquitoes in their adult stages based on their morphological characters requires multiple body parts, including the abdominal terga and wing, to aid in decision making [5,7]. However, it is difficult to identify them in the case of damaged specimens when some of their morphological character is missing due to transit or gathering in the field [8]. Presently, many modern techniques have emerged to assist medical entomologists in identifying specimens, such as GMs and DNA barcoding [9,10,11].

The geometric morphometric (GM) technique is a useful modern approach to investigate differences in wing shape for species classification when some species of mosquitoes are difficult to identify by standard taxonomic methods [12,13,14]. In addition, this approach has been used to evaluate the morphological changes caused by environmental conditions in insect vector populations [15,16,17,18,19,20,21,22]. Previously, this technique was applied to identify cryptic species of the *Anopheles dirus* and the *An. barbirostris* complexes in Thailand, and it was found that GM had a high level of discriminating efficiency in many member species [23,24]. In addition, this modern technique was also proven to be effective with several insects of medical and veterinary significance [25,26,27,28,29]. However, the success of this technique is not guaranteed for all mosquito species, which depends on sufficient differences in geometrical shapes between species [30]. Phanitchakun et al. (2019)’s earlier study revealed that four *Lutzia* species in Thailand had different wing vein structures, especially between *Lt. chiangmaiensis* and *Lt. vorax* [4]. Therefore, it is possible that the GM technique could help identify member species in the genus *Lutzia* in Thailand. Ruangsittichai et al. [31] explained that the application of GM techniques to particular species for the first time should be supported by molecular biology techniques. DNA barcoding is a recognized effective choice in supporting and confirming the results of GM [31,32,33]. In addition, DNA barcoding sequences of several mosquito species were recently collected across Thailand, which could be used to better confirm the results of GM [3].

Therefore, in the present study, we aimed to evaluate the efficacy of the landmark-based GM approach for the identification of mosquito species within the genus *Lutzia* in Thailand: *Lt. chiangmaiensis*, *Lt. fuscana*, *Lt. halifaxii*, and *Lt. vorax*. In addition, DNA barcoding based on a *COI* sequence analysis was also used in parallel with the landmark-based GM approach to identify four *Lutzia* species. The results of this study can provide a novel method to more efficiently identify mosquitoes in the genus *Lutzia*.

## 2. Materials and Methods

### 2.1. Mosquito Collection 

**Figure 1:**
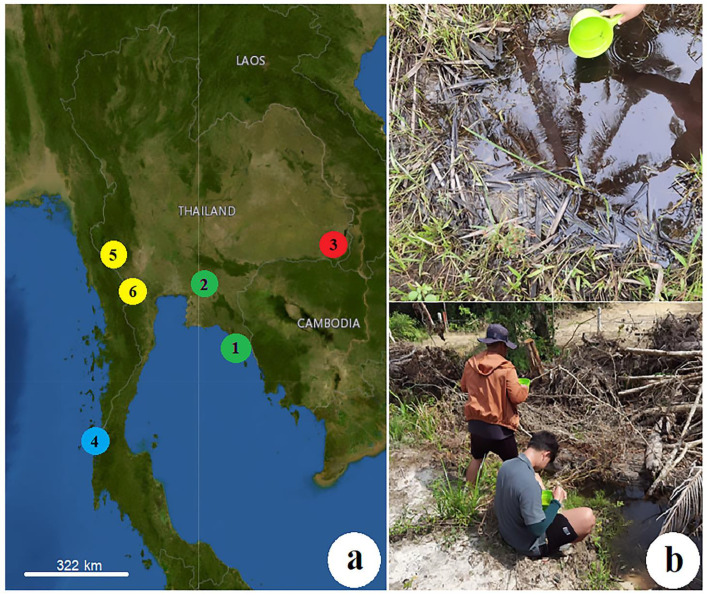
Locations of the sample collection sites (**a**) and *Lutzia* larval collection method used in this study (**b**). *Lutzia* mosquito samples were gathered from six provinces in four geographical regions of Thailand, including Trat (1), Chachoengsao (2) in the eastern region (green), Ubon Ratchathani (3) in the northeastern (red), Ranong (4) in the southern region (blue), Kanchanaburi (5), and Ratchaburi (6) in the western region (yellow). This map was made available by the USGS National Map Viewer (public domain): http://viewer.nationalmap.gov/viewer/, accessed on 1 October 2022.

All *Lutzia* larvae were collected from various breeding sites in six provinces of Thailand: Trat (eastern Thailand; 12°08′27.9″ N, 102°16′35.1″ E and 11°38′16.3″ N, 102°33′23.0″ E), Chachoengsao (eastern Thailand; 13°27′14.0″ N, 101°46′25.5″ E), Ubon Ratchathani (northeastern Thailand; 14°34′38.6″ N, 105°21′41.2″ E), Ranong (southern Thailand; 9°50′28.5″ N, 98°27′15.6″ E and 9°43′47.4″ N, 98°23′56.1″ E), Kanchanaburi (western Thailand; 14°06′02.5″ N, 99°00′01.1″ E), and Ratchaburi (western Thailand; 13°22′34.0″ N, 99°16′26.0″ E) between June 2021 and April 2022 (Figure 1). 

**Figure 2:**
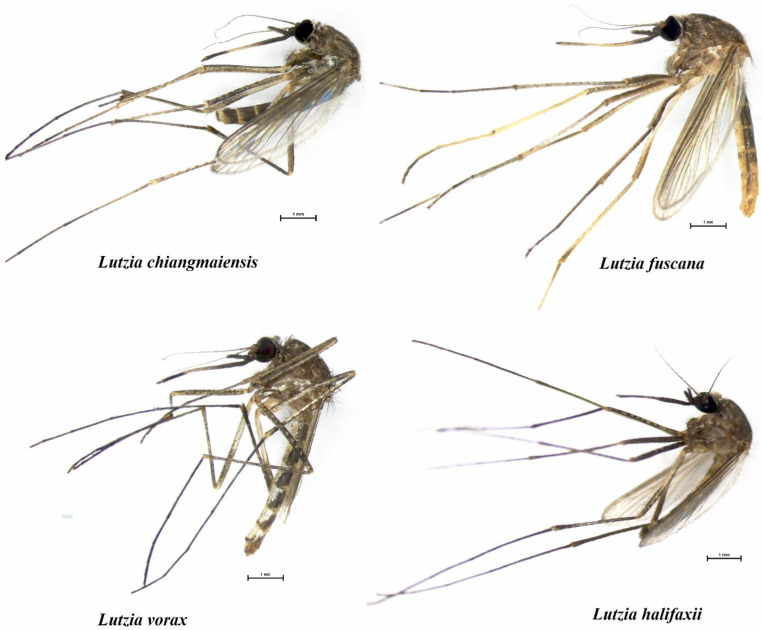
Four adult female *Lutzia* species used in the study: *Lt. chiangmaiensis*, *Lt. fuscana*, *Lt. halifaxii*, and *Lt. vorax*. *Lutzia fuscana*, *Lt. halifaxii*, and *Lt. vorax* have different abdominal terga characteristics: *Lt. fuscana* has entirely pale light-yellow scales on the terga V–VIII and entirely dark scales or narrow apical pale bands on the terga II–IV; *Lt. halifaxii* has fully covered dark scales on the abdominal terga and occasional lateral pale patches on the last few segments; *Lt. vorax* has apical pale stripes that are rather wide and around the same width; and *Lt. chiangmaiensis* has abdominal terga characteristics like those of *Lt. vorax* but with different wing vein positions.

The mosquito larvae were transported to the College of Allied Health Sciences at the Suan Sunandha Rajabhat University, Samut Songkhram, Thailand, where they were raised in white plastic trays (20 larvae per tray) in the biology laboratory at 25–28 °C, a 12–12 h light–dark cycle, and 50–60% relative humidity until they reached the pupal stage. Third-stage *Aedes* larvae from the field were used as food for the *Lutzia* larvae, with each tray receiving 50 larvae daily. When the larvae became pupae, they were relocated to a small cup containing clean water and placed in 30 × 30 × 30 cm cages to facilitate the collection of adult mosquitoes. Four mature female *Lutzia* species (*Lt. chiangmaiensis*, *Lt. fuscana*, *Lt. halifaxii*, and *Lt. vorax*; Figure 2) were identified based on their physical characteristics under a stereomicroscope using illustrated taxonomic keys to the mosquitoes of Thailand after being euthanized in the freezer at –20 °C [5,7,34].

### 2.2. Geometric Morphometrics

After the morphological identification, the right wings of the four *Lutzia* species were cut off from their thorax by fine forceps and mounted using Hoyer’s mounting medium on glass microscope slides with coverslips. A digital camera (Nikon DS-Fi3, Tokyo, Japan) linked to a Nikon SMZ 800 N stereomicroscope (Nikon Corp., Tokyo, Japan) was used to take photos of all the mounted wing slides of the *Lutzia* samples, adding a 1 mm scale bar to each wing image.

**Figure 3:**
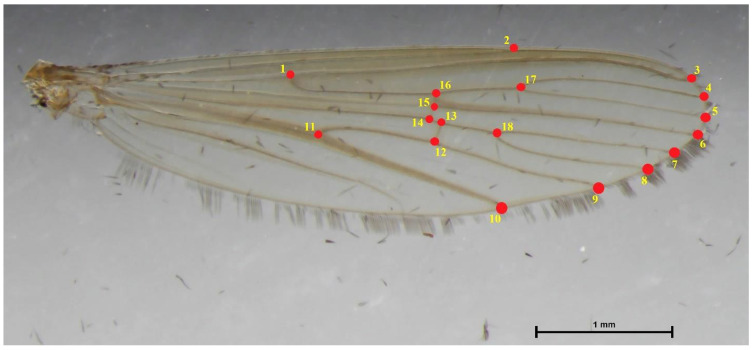
Eighteen landmarks on the wing vein structure of *Lutzia* mosquito for GM analyses in this study.

Eighteen landmarks on the wing vein structure of the *Lutzia* mosquitoes were identified and digitized for the GM analyses (Figure 3). This study’s landmark placements are based on earlier research that successfully identified certain mosquito species [23,24,35]. 

In order to evaluate the accuracy of landmark plotting in a wing image set, also known as a repeatability test, 10 images from each *Lutzia* species were chosen at random and re-digitized by the same individual. The Procrustes analysis was used to estimate shape repeatability [36]. In addition, the linear determination coefficient was calculated after regressing the wing size on the wing shape, in order to assess allometry, which is the relationship between wing size and wing shape [30].

Wing shape variables were computed by the Generalized Procrustes Analysis. The shape matrix was held in Euclidean space to construct partial warps, wherein the principal components of the partial warps were used as the final shape variables [37]. After that, the final shape variables were used for various statistical calculations. The discriminant analysis was used to examine the group separation among the *Lutzia* species, which is represented as a discriminant space (also called a factor map), and to compute the Mahalanobis distance for assessing shape divergence between the *Lutzia* species. 

The significant difference in wing shape between four *Lutzia* species based on the pairwise Mahalanobis distance was performed using a non-parametric test (1000 permutations) with Bonferroni correction at *p* < 0.05. 

To evaluate wing shape similarity between the four *Lutzia* species, a hierarchical clustering tree was constructed based on Mahalanobis distances. Finally, Mahalanobis-based validated (cross-checked) classification was used to evaluate the efficacy of species identification based on wing shape variables, with each sample sequentially removed from the total sample and assigned to the closest group, performing this for all mosquito samples.

### 2.3. DNA Extraction, PCR Amplification, and DNA Sequencing

Ten *Lutzia* mosquitoes were randomly sampled per species (a total of 40 samples) for DNA extraction. The total genomic DNA of the mosquitoes was extracted from 4 to 6 legs of each adult *Lutzia* specimen, using FavorPrep™ Mini Kits (Favorgen Biotech, Ping-Tung, Taiwan), following the manufacturer’s guidelines. Both universal barcode primers, including forward (5′-GGA TTT GGA AAT TGA TTA GTT CCT T-3′) and reverse (5′-AAA AAT TTT AAT TCC AGT TGG AAC AGC-3′) primers [38], were used to amplify a 709-bp fragment of *COI*; the polymerase chain reaction (PCR) reaction mixture and PCR conditions were as described in a previous study [3]. Negative (water without DNA) and positive controls (DNA of *Lutzia* mosquitoes) were included in each PCR. All PCR products were examined through electrophoresis using 1.5% agarose gels and Tris-borate-EDTA (TBE) buffer, as well as staining with the Midori Green DNA stain (Nippon Gene, Tokyo, Japan), and visualized via the ImageQuant LAS 500 imager (GE Healthcare Japan Corp., Tokyo, Japan). After that, quality PCR products were purified and sequenced, in both forward and reverse, by SolGent, Inc. (Daejeon, Republic of Korea).

### 2.4. Software

For the GM analyses, the online XYOM version 2 was used in this study [39]. While for the molecular analyses, the trace files of the *COI* sequences were examined and manually edited using the BioEdit software [40]. Both forward and reverse sequences were used to create a consensus sequence using the BioEdit software. Our consensus sequences were compared to DNA sequences available in the GenBank database of the National Center for Biotechnology Information website (https://blast.ncbi.nlm.nih.gov/Blast.cgi/, accessed on 5 October 2022) and the Barcode of Life Database (BOLD) available at https://www.boldsystems.org/index.php/IDS_OpenIdEngine, accessed on 5 October 2022 to identify the *Lutzia* species. 

The multiple sequence alignment of the *COI* sequences of the *Lutzia* mosquitoes were performed using Clustal W software [41] in MEGA X [42]. The nucleotide composition and genetic divergences (within and between the *Lutzia* species) were calculated by the Kimura-2 parameter (K2P) model via MEGA X [42]. A neighbor-joining (NJ) tree based on the K2P distances with 1000 bootstraps was constructed using MEGA X [42] to examine the genetic relationship between the *Lutzia* species.

## 3. Results

### 3.1. Lutzia Species

In this study, 179 individuals of the four *Lutzia* species were gathered. The morphological identification of the *Lutzia* specimens grouped them into four species: *Lt. chiangmaiensis*, *Lt. fuscana*, *Lt. halifaxii*, and *Lt. vorax* (Table 1). Most of the collected *Lutzia* samples were identified as *Lt. fuscana* (58 individuals, 32.40%), followed by *Lt. halifaxii* (47 individuals, 26.26%), *Lt. chiangmaiensis* (45 individuals, 25.14%), and *Lt. vorax* (29 individuals, 16.20%), respectively. Based on our survey, all four *Lutzia* species were collected in Trat, eastern Thailand, whereas in Ranong, southern Thailand, only *Lt. halifaxii* was found. 

### 3.2. Wing Geometric Morphometrics

**Figure 4:**
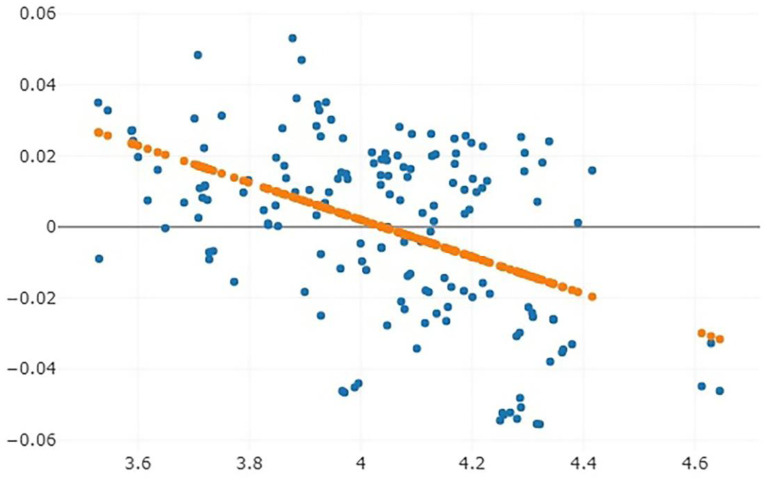
Allometric relationship between the wing centroid size (CS) and wing shape of *Lutzia* mosquito specimens. The linear regression prediction is shown by the orange dotted line; the y-axis shows the wing shape, and the x-axis shows the wing CS.

A total of 157 undamaged *Lutzia* wings were utilized for the GM analyses: 40 wings of *Lt. chiangmaiensis*, 50 wings of *Lt. fuscana*, 42 wings of *Lt. halifaxii*, and 25 wings of *Lt. vorax*. Assessing the repeatability of landmark digitizing on wing image sets revealed a high degree of shape repeatability based on the Procrustes analysis (repeatability percentage score = 96%; measurement error percentage score = 4%). The repeatability result indicated that landmark digitization in the tested wing image set showed a high accuracy rate, while investigating the allometry revealed a significant relationship between the wing size and wing shape of the *Lutzia* mosquitoes. The linear determination coefficient after regression showed a negative correlation between size and shape. The effect of wing size (wing centroid size) on wing shape (the discriminant factor) based on the linear determination coefficient was 22% (r^2^) (*p* < 0.05) (Figure 4).

**Figure 5:**
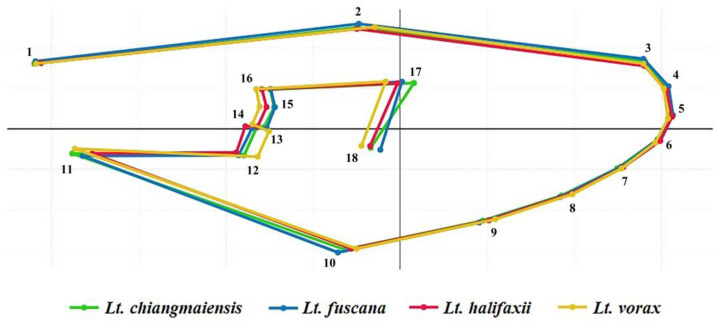
Superimposed wireframe graph showing the superposition of the average wing shape of each *Lutzia* species, including *Lt. chiangmaiensis*, *Lt. fuscana*, *Lt. halifaxii*, and *Lt. vorax*.

**Figure 6:**
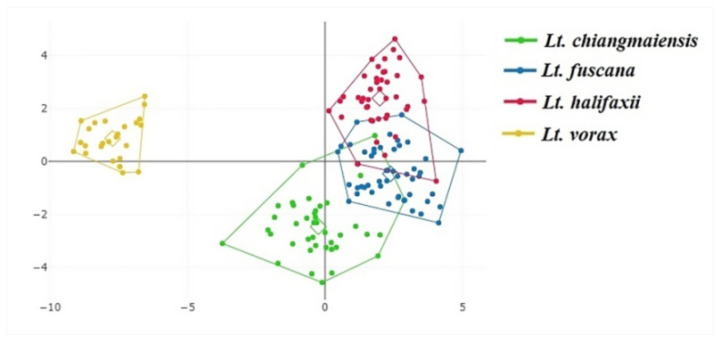
Discriminant space produced by the discriminant analysis of the wing shape of *Lt. chiangmaiensis*, *Lt. fuscana*, *Lt. halifaxii*, and *Lt. vorax*. Four *Lutzia* species’ wing shape variations are represented by each polygon, and each dot inside the polygons represents each individual sample.

**Figure 7:**
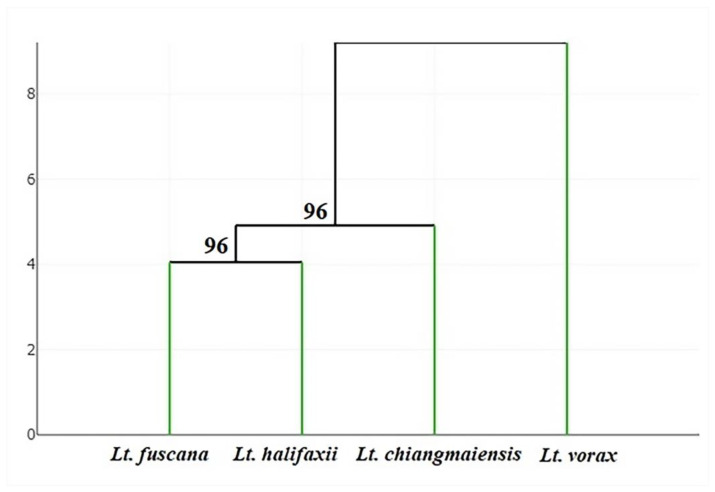
Hierarchical clustering tree based on the Mahalanobis distances between average group shapes showing wing shape similarity among *Lutzia* species. The percentages of bootstrap values based on 1000 replicates are shown above the branches.

The superposition of the average wing shapes revealed shape variation differences among the four *Lutzia* species, especially at landmark positions 12, 13, 17, and 18 (Figure 5). Investigating the wing shape based on the final shape variables by discriminant analysis displayed that the *Lt. vorax* group was clearly separated from the other *Lutzia* groups, whereas some specimens of the *Lt. chiangmaiensis*, *Lt. fuscana*, and *Lt. halifaxii* groups overlapped with each other (Figure 6). Pairwise Mahalanobis distances, which were used to investigate the wing shape differences between the species, were significantly different in all *Lutzia* species pairs (*p* < 0.05, Table 2). Wing shape similarity between the four *Lutzia* species was visualized by a hierarchical clustering tree based on the Mahalanobis distances (Figure 7). 

The efficacy of the landmark-based GM approach based on wing shape in identifying the *Lutzia* species was evaluated by a cross-validated reclassification test, which is shown in Table 3. The results of the reclassification test clearly indicated that wing shape yielded a high level of successful identification (correct identifications ranged from 92.50% to 100%). *Lutzia vorax* had the highest correct classification score (100%), while *Lt. chiangmaiensis* had the least correct classification score (92.50%). 

### 3.3. Barcode Sequences

Using the GenBank database and the BOLD system for preliminary species identification, the *COI* sequences of *Lt. chiangmaiensis* and *Lt. vorax* obtained in our study were correctly identified (>99% similarity), while a more than 99% similarity overlap between *Lt. fuscana* and *Lt. halifaxii* was found from our sequence comparison with available species sequences in the GenBank database and the BOLD system. 

The average nucleotide compositions of the entire dataset of the 40 *Lutzia* mosquito sequences were A (29.9%), T (38.8%), G (15.4%), and C (15.9%). The absence of stop codons in the amino acid translations indicated that all sequences were functioning protein-coding genes and not pseudogenes. 

The intraspecific genetic divergence (within species) based on the K2P model of the *Lutzia* species varied from 0.00% to 1.43%, and the average intraspecific divergence was 0.48% (Table 4). The highest average intraspecific divergence was observed in *Lt. vorax* (0.76%), followed by *Lt. fuscana* (0.60%), *Lt. halifaxii* (0.35%), and *Lt. chiangmaiensis* (0.19%), respectively. The interspecific genetic divergence (between species) of the *Lutzia* species varied from 0.00% to 5.60%, and the average interspecific divergence was 3.14%. The highest average interspecific divergence was observed between *Lt. vorax* and *Lt. chiangmaiensis* (5.11%), followed by that between *Lt. vorax* and *Lt. fuscana* (4.96%), and between *Lt. vorax* and *Lt. halifaxii* (4.74%). Meanwhile, the lowest average interspecific divergence was observed between *Lt. fuscana* and *Lt. halifaxii* (0.48%). The results of the K2P distances showed the overlap between intra- and interspecific divergence between *Lt. halifaxii* and *Lt. fuscana*.

**Figure 8:**
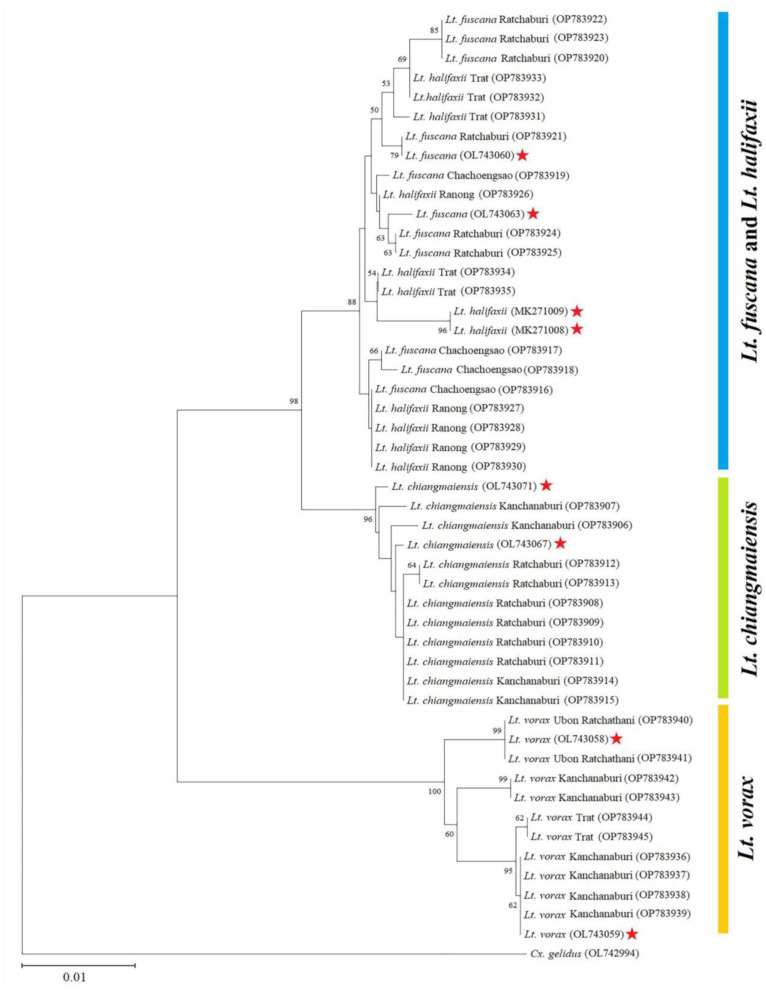
Neighbor-joining (NJ) phylogenetic tree based on Kimura 2-parameter (K2P) distances derived from the 40 *COI* barcoding sequences of four *Lutzia* species. Eight sequences from the GenBank database (accession numbers OL743067 and OL743071 for *Lt. chiangmaiensis*; OL743060 and OL743063 for *Lt. fuscana*; MK271008 and MK271009 for *Lt. halifaxii*; OL743058 and OL743059 for *Lt. vorax*) were used as reference species sequences (red stars)*. Culex gelidus* (accession number: OL743067) was used as an outgroup. The bootstrap values (1000 replicates) below 50% are not shown.

A neighbor-joining phylogenetic analysis revealed three distinct *Lutzia* clusters, including the *Lt. chiangmaiensis* cluster (bootstrap value = 96%), the *Lt. fuscana* and *Lt. halifaxii* cluster (bootstrap value = 88%), and the *Lt. vorax* cluster (bootstrap value = 100%) (Figure 8). This result strongly visualized that *Lt. fuscana* and *Lt. halifaxii* samples were grouped into the same cluster based on similarity with the sequences of both mosquito species.

The *COI* barcode sequence data of the four obtained *Lutzia* species were submitted to the GenBank database under the following accession numbers: *Lt. chiangmaiensis* (OP783906–OP783915), *Lt. fuscana* (OP783916–OP783925), *Lt. halifaxii* (OP783926–OP783935), and *Lt. vorax* (OP783936–OP783945). 

## 4. Discussion

In the present study, we evaluated the efficacy of the landmark-based GM approach coupled with DNA barcoding based on *COI* sequence analysis to identify four *Lutzia* species in Thailand: *Lt. chiangmaiensis*, *Lt. fuscana*, *Lt. halifaxii*, and *Lt. vorax*. A total of 179 *Lutzia* mosquitoes, consisting of 45 individuals of *Lt. chiangmaiensis*, 58 individuals of *Lt. fuscana*, 47 individuals of *Lt. halifaxii*, and 29 individuals of *Lt. vorax*, were collected from six provinces of Thailand: Trat, Chachoengsao, Ubon Ratchathani, Ranong, Kanchanaburi, and Ratchaburi. 

Rattanarithikul et al. [5] reported that *Lt. fuscana* and *Lt. halifaxii* are distributed throughout Thailand, whereas the distribution of *Lt. vorax* in the southern, northeastern, and eastern regions is unclear. Our survey revealed the presence of *Lt. vorax* in the northeastern (Ubon Ratchathani) and eastern (Trat and Chachoengsao) regions of Thailand. Furthermore, *Lt. chiangmaiensis* is the newest *Lutzia* species in Thailand, which was discovered in northern Thailand by Somboon and Harbach [7]. This species was reported only in northern Thailand. This study reported the existence of *Lt. chiangmaiensis* for the first time in other regions of Thailand, including the eastern (Trat and Chachoengsao), northeastern (Ubon Ratchathani), and western (Kanchanaburi and Ratchaburi) regions.

At present, the standard morphological method for identifying mosquito species is recognized as error-prone and requires practitioners with extensive experience [43]. Nonetheless, this method is highly accepted when supported and confirmed by effective alternative or complementary techniques [8]. 

Our findings indicated that the landmark-based GM approach based on wing shape analyses was very successful in identifying *Lutzia* species in Thailand, which is supported by a high percentage of correct classification (94.27% of total performance). Correctly classified specimens of *Lt. chiangmaiensis* (92.50%), *Lt. fuscana* (94%), *Lt. halifaxii* (92.86%), and *Lt. vorax* (100%) yielded a high percentage of species identification success. The observation of the wireframe graph based on the superposition of the average wing shape revealed differences in wing structure between the four *Lutzia* species. According to previous research, the wing vein pattern of many mosquito species is a species-specific identity that may be detected by the landmark-based GM for identification [10,13,44,45,46]. The results of the discriminant analysis, pairwise Mahalanobis distance, and 100% identification success based on the cross-validated reclassification indicated that the wing shape of *Lt. vorax* differed markedly from that of the other *Lutzia* species. GM’s results are consistent with previous studies of Phanitchakun et al. [4] and Somboon and Harbach [7], reporting that the wing of *Lt. vorax* has the mediocubital crossvein situated distal to the radiomedial crossvein, unlike other *Lutzia* species.

In this study, wing size was not analyzed for the identification of the four *Lutzia* species. Almost all previous studies showed a failure to identify mosquito species by the landmark-based GM approach based on wing size analyses. The wing size of mosquitoes is not a conserved trait and will fluctuate from generation to generation according to density-dependent and independent selective pressures present during the immature stage of development [30,31]. In addition, Lorenz et al. [30] explained that the wing size is more sensitive to a changing environment and frequently overlaps among species, which is difficult to interpret. 

An examination of the allometry indicated the relation between the wing size and wing shape of the *Lutzia* mosquito samples. The result of linear regression for allometric estimation showed a negative correlation (also called an inverse correlation), which means size decreases as shape difference increases, or size increases as shape difference decreases (one increases as the other decreases). This relationship pattern of *Lutzia* mosquitoes is similar to those found in studies that examined three cryptic species of the *An. barbirostris* complex in Thailand [24]. Nevertheless, this correlation has no effect on species identification (inter-species investigations) based on the wing shape analyses [47].

Furthermore, the limitation of our study is that larvae were collected and reared into adults for species investigations. It is difficult for adult *Lutzia* mosquitoes to be collected from mosquito traps and using the human landing catch method, as these methods do not attract them [5]. The preferred method of collecting specimens of mosquitoes in the genus *Lutzia* is the larval collection in breeding sites such as rice fields, small ponds, roadside ditches, shallow wells, bamboo cups, small puddles, water jars, and wheel tracks [4,5]. Therefore, the GM results of this study may differ from adult specimens in nature due to the influence of factors acquired during the development of the immature stage. However, environmental influences during the development of the immature stage of mosquitoes tend to affect size more than shape in the adult stage [31], while wing shape is relatively stable due to being influenced by the genetic background [30]. In this study, the identification of *Lutzia* species based on GM offers good results for the wing shape analysis and may be a potential alternative for future applications in the field. For more effective results in applications, we recommend that GM performance be tested on *Lutzia* specimens in new study areas to avoid errors from wing shape variations in different sites.

This is the first application of the landmark-based GM approach to identify mosquito species in the genus *Lutzia.* Thus, DNA barcoding based on the *COI* sequence analysis was used to identify *Lutzia* species coupled with the GM approach. These genetic results indicated that *COI* barcoding could distinguish only two of the four *Lutzia* species, namely, *Lt. chiangmaiensis* and *Lt. vorax*, as supported by the barcoding gap and the NJ phylogenetic analysis. Due to their low interspecific differences, it is impossible to distinguish between *Lt. fuscana* and *Lt. halifaxi*. The barcoding gap is a hiatus of difference between the greatest intraspecific genetic distance and the smallest interspecific distance, which is very important in determining the success of DNA barcoding [3]. Our assessment of genetic divergences revealed that this gap was not present between *Lt. fuscana* and *Lt. halifaxii*, indicating that the DNA barcoding method could not discriminate between the two species. This result is consistent with the investigation of Phanitchakun et al. [4], which found that *Lt. fuscana* and *Lt. halifaxii* were not clearly distinct in *COI* and *COII* sequences. We also found that *Lt. chiangmaiensis* was genetically more closely related to *Lt. fuscana* and *Lt. halifaxii*, with low genetic differences between species groups (1.86% and 1.70% average interspecific genetic divergences, respectively). The results of genetic divergences based on the K2P model were consistent with the result of our phylogenetic tree.

To confirm that the failure of DNA barcoding to identify *Lt. fuscana* and *Lt. halifaxii* was not attributable to faulty morphological identification, we compared all of our sequence samples to those *Lutzia* sequences available in public databases. The comparison results indicated that almost all overlap between *Lt. fuscana* and *Lt. halifaxii* in the database was observed. Therefore, the findings of this research concluded that DNA barcoding is not an effective approach for differentiating *Lutzia* species in Thailand. Recently, DNA barcoding was applied to aid mosquito identification in Thailand and found that although this technique has high efficiency, some mosquitoes are unable to identify the exact species, such as *Anopheles dirus* and *An. baimaii* [23].

## 5. Conclusions

In this study, we evaluated the efficacy of modern techniques, including the landmark-based GM approach and DNA barcoding, to support the standard morphological method for the identification of *Lutzia* species in Thailand. Our results showed that DNA barcoding was poorly effective in identifying *Lt. fuscana* and *Lt. halifaxii* based on a lack of barcoding gap. In contrast, the GM approach based on wing shape analyses successfully identified four *Lutzia* species in Thailand. Therefore, GM can help medical entomologists to identify their species in the field. Compared to molecular biology techniques, the GM approach is less expensive and does not require advanced scientific equipment. However, it is difficult to prepare wing slides for this approach, despite the rapid speed of analysis. In addition, our survey results update the distribution of *Lt. chiangmaiensis* and *Lt. vorax* in Thailand. This research will help medical entomologists to more efficiently identify mosquitoes in the genus *Lutzia*, resulting in more effective mosquito control and surveillance.

## Figures and Tables

**Table 1:** Number of *Lutzia* mosquitoes collected in this study.

Province	Total Number of *Lutzia* Mosquitoes Collected			
	*Lt. chiangmaiensis*	*Lt. fuscana*	*Lt. halifaxii*	*Lt. vorax*
Eastern Thailand				
Trat	8	13	32	6
Chachoengsao	5	30	–	4
Northeastern Thailand				
Ubon Ratchathani	5	–	–	4
Southern Thailand				
Ranong	–	–	15	–
Western Thailand				
Kanchanaburi	15	–	–	5
Ratchaburi	12	15	–	10
Total	45	58	47	29

**Table 2:** Pairwise Mahalanobis distance and significant differences in the wing shape of *Lt. chiangmaiensis*, *Lt. fuscana*, *Lt. halifaxii*, and *Lt. vorax*.

*Lutzia* Species	Pairwise Mahalanobis Distance			
	*Lt. chiangmaiensis*	*Lt. fuscana*	*Lt. halifaxii*	*Lt. vorax*
*Lt. chiangmaiensis*	0.00			
*Lt. fuscana*	4.55 *	0.00		
*Lt. halifaxii*	5.34 *	4.05 *	0.00	
*Lt. vorax*	8.45 *	10.25 *	9.99 *	0.00

Comparing the wing shape between species, superscript asterisks after pairwise Mahalanobis distance values indicate statistically significant differences between *Lutzia* species at *p*-value < 0.05.

**Table 3:** Identification error and percentage of correct classification based on the wing shape of *Lt. chiangmaiensis*, *Lt. fuscana*, *Lt. halifaxii*, and *Lt. vorax* calculated by a cross-validated reclassification test.

*Lutzia* Species	Classified as				Total (Individuals)	Correct Identifications (%)
	*Lt. chiangmaiensis* (Individuals)	*Lt. fuscana* (Individuals)	*Lt. halifaxii* (Individuals)	*Lt. vorax* (Individuals)		
*Lt. chiangmaiensis*	37	1	2	0	40	92.50%
*Lt. fuscana*	0	47	3	0	50	94%
*Lt. halifaxii*	1	2	39	0	42	92.86%
*Lt. vorax*	0	0	0	25	25	100%

**Table 4:** Average percentage intra- and interspecific K2P distances of four *Lutzia* species based on the *COI* barcoding sequences calculated using the Kimura 2-parameter distance algorithm.

*Lutzia* Species	Average Percentage Genetic Divergences (Min–Max)			
	*Lt. chiangmaiensis*	*Lt. fuscana*	*Lt. halifaxii*	*Lt. vorax*
*Lt. chiangmaiensis*	0.19% (0.00–0.71)			
*Lt. fuscana*	1.86% (1.43–2.45)	0.60% (0.00–1.14)		
*Lt. halifaxii*	1.70% (1.57–2.16)	0.48% (0.00–0.85)	0.35% (0.00–0.57)	
*Lt. vorax*	5.11% (4.81–5.44)	4.96% (4.22–5.60)	4.74% (4.38–5.29)	0.76% (0.00–1.43)

The red values are intraspecific genetic distances, while the black values are the interspecific genetic distances.

## Data Availability

The *COI* sequence data obtained in this study were deposited in the GenBank database, and the accession numbers are as follows: *Lutzia chiangmaiensis*: OP783906–OP783915; *Lutzia fuscana*: OP783916–OP783925; *Lutzia halifaxii*: OP783926–OP783935; *Lutzia vorax*: OP783936–OP783945.
